# Use of an allostatic neurotechnology by adolescents with postural orthostatic tachycardia syndrome (POTS) is associated with improvements in heart rate variability and changes in temporal lobe electrical activity

**DOI:** 10.1007/s00221-015-4499-y

**Published:** 2015-12-08

**Authors:** John E. Fortunato, Catherine L. Tegeler, Lee Gerdes, Sung W. Lee, Nicholas M. Pajewski, Meghan E. Franco, Jared F. Cook, Hossam A. Shaltout, Charles H. Tegeler

**Affiliations:** Virginia Commonwealth University, Richmond, VA USA; Hypertension and Vascular Research Center, Wake Forest School of Medicine, Winston-Salem, NC USA; Department of Neurology, Wake Forest School of Medicine, Winston-Salem, NC USA; Brain State Technologies LLC, Scottsdale, AZ 85260 USA; Department of Biostatistical Sciences, Wake Forest School of Medicine, Winston-Salem, NC USA; Departments of Obstetrics and Gynecology and General Surgery, Wake Forest School of Medicine, Winston-Salem, NC USA

**Keywords:** POTS, Autonomic dysregulation, Nausea, Allostasis, Neurotechnology, Hemispheric asymmetry

## Abstract

Autonomic dysregulation and heterogeneous symptoms characterize postural orthostatic tachycardia syndrome (POTS). This study evaluated the effect of high-resolution, relational, resonance-based, electroencephalic mirroring (HIRREM^®^), a noninvasive, allostatic neurotechnology for relaxation and auto-calibration of neural oscillations, on heart rate variability, brain asymmetry, and autonomic symptoms, in adolescents with POTS. Seven subjects with POTS (three males, ages 15–18) underwent a median of 14 (10–16) HIRREM sessions over 13 (8–17) days. Autonomic function was assessed from 10-min continuous heart rate and blood pressure recordings, pre- and post-HIRREM. One-minute epochs of temporal high-frequency (23–36 Hz) brain electrical activity data (T3 and T4, eyes closed) were analyzed from baseline HIRREM assessment and subsequent sessions. Subjects rated autonomic symptoms before and after HIRREM. Four of seven were on fludrocortisone, which was stopped before or during their sessions. Heart rate variability in the time domain (standard deviation of the beat-to-beat interval) increased post-HIRREM (mean increase 51 %, range 10–143, *p* = 0.03), as did baroreflex sensitivity (mean increase in high-frequency alpha 65 %, range −6 to 180, *p* = 0.05). Baseline temporal electrical asymmetry negatively correlated with change in asymmetry from assessment to the final HIRREM session (*p* = 0.01). Summed high-frequency amplitudes at left and right temporal lobes decreased a median of 3.8 μV (*p* = 0.02). There was a trend for improvements in self-reported symptoms related to the autonomic nervous system. Use of HIRREM was associated with reduced sympathetic bias in autonomic cardiovascular regulation, greater symmetry and reduced amplitudes in temporal lobe high-frequency electrical activity, and a trend for reduced autonomic symptoms. Data suggest the potential for allostatic neurotechnology to facilitate increased flexibility in autonomic cardiovascular regulation, possibly through more balanced activity at regions of the neocortex responsible for autonomic management.

*Clinical trial registry* “Tilt Table with Suspected postural orthostatic tachycardia syndrome (POTS) Subjects,” Protocol Record: WFUBAHA01.

## Introduction

The pervasive role of the autonomic nervous system for regulating functionality across organ systems and behaviors is often underappreciated in both adult and pediatric medicine (Rees 2014). For example, unexplained gastrointestinal symptoms are common in children (Hyams et al. 1996), and autonomic testing for children with functional abdominal pain can reveal differences in pathophysiology that may be indicative of an underlying dysautonomia (Safder et al. 2009). One such dysautonomia is postural orthostatic tachycardia syndrome, or POTS, defined as excessive increase in heart rate (HR) upon upright posture (Freeman et al. 2011; Ojha et al. 2011). POTS may present with heterogeneous symptoms including palpitations, shortness of breath, sleep disturbance, fatigue, nausea, abdominal pain, headache, lightheadedness, or syncope. Treatment may include agents intended to remediate dysregulation across cardiovascular, gastrointestinal, and neuropsychiatric systems, including mineralocorticoids or saline for volume expansion, beta-blockers and other autonomic medications, and others (Raj et al. 2005; Garland et al. 2007). Yet the genesis of POTS symptomatology is likely multifactorial involving numerous descending structures of the central autonomic network including the brainstem, amygdala, insula, anterior cingulate cortex, hypothalamus, periaqueductal gray, and others, and there is an important role for behavioral or other interventions that can impact this network systematically on a top-down basis (Benarroch 2012).

Studies have shown that at the level of the cerebral hemispheres, regulation of the autonomic nervous system (ANS) is lateralized, with the right and left sides being associated with sympathetic and parasympathetic management, respectively (Zamrini et al. 1990; Oppenheimer et al. 1992; Yoon et al. 1997; Hilz et al. 2001; Lee et al. 2014). Furthermore, there is evidence that it may be possible to detect asymmetrical contributions of the cerebral hemispheres to management of the autonomic nervous system through the use of noninvasive scalp recordings. Changes in cortical potential measured from the left temporal scalp have been shown to correlate with measures of cardiac function (Gray et al. 2007), and rightward dominance in temporal high-frequency brain electrical asymmetry calculated from scalp measures has been shown to correspond to higher resting heart rate and lower baroreflex sensitivity in a heterogeneous population (Tegeler et al. 2015b). Neurotechnology interventions have been shown to have a role for modulating autonomic activity through engagement with patterns of lateralized activity in cortical regions associated with ANS management. Transcranial direct current stimulation at the left temporal lobe, for example, is an “open-loop” approach (one that provides stimulus without recording brain activity) that has been applied at the left temporal region in order to augment parasympathetic functioning and potentially enhance performance in cyclists (Okano et al. 2015).

The potential contribution of hemispheric lateralization patterns to autonomic dysregulation in POTS has not previously been explored. High-resolution, relational, resonance-based, electroencephalic mirroring (HIRREM^®^) is a novel, noninvasive closed-loop neurotechnology designed to facilitate relaxation and auto-calibration of neural oscillations. HIRREM monitors brain electrical activity at high spectral resolutions using two-channel recordings (Gerdes et al. 2013). It applies software algorithms to generate unique and changing patterns of acoustic stimulation (tones of variable pitch and timing) that are based on real-time changes in dominant frequencies of brain electrical activity. In a randomized, controlled pilot trial for individuals with insomnia, use of HIRREM was associated with improved sleep and reduced depressive symptoms (Tegeler et al. 2012), and in an open-label study of patients with heterogeneous symptoms, use of HIRREM was associated with reduction in temporal lobe high-frequency electrical asymmetry and increased heart rate variability (Tegeler et al. 2013).

Presently, we report on the use of HIRREM in a series of adolescents with nausea and orthostatic symptoms who were diagnosed with POTS by an abnormal head upright tilt (HUT) test. Specifically, we examined pre- to post-HIRREM change in outcome measures of autonomic cardiovascular regulation including: heart rate variability as measured by standard deviation of the beat-to-beat interval (SDNN), baroreflex sensitivity (BRS), asymmetry in temporal lobe high-frequency (23–36 Hz) electrical activity, the sum of right and left temporal high-frequency electrical amplitudes, and autonomic symptom scores.

## Methods

### Subject recruitment and selection

Subjects for this study were recruited from the pediatric gastroenterology clinic of Wake Forest Baptist Medical Center, in whom evaluation of chronic unexplained nausea and orthostatic symptoms (dizziness, flushing, tachycardia, shortness of breath with standing) revealed an abnormal cardiovascular response to HUT. We specifically included only subjects meeting criteria for POTS (Fortunato et al. 2011). A convenience sample of seven of these patients (three male, ages 15–18, one Asian, one African-American) was invited to enroll in an ongoing open-label, single-site, IRB-approved, feasibility study of HIRREM for individuals with diverse psychophysiological conditions, conducted by the Department of Neurology. Informed consent was obtained from all individual participants (18 years of age or greater) included in the study. For those subjects less than 18 years of age, a study consent was obtained from the patient’s parent or legal guardian, as well as a separate assent from the patient. Four of the seven subjects were taking fludrocortisone prior to enrollment, which was discontinued before or during HIRREM in all four.

### Head upright tilt (HUT) table test

The tilt table evaluation was done using standard methods, previously reported (Fortunato et al. 2011). After 15-min supine, there was an immediate upright tilt from 0° to 70°. The table remained at 70° for 45 min, before being returned to supine for another 15 min. Pulse oximetry and HR were monitored continuously, with blood pressure (BP) monitored every 2 min. POTS was defined as either a HR of greater than 120 beats per minute (bpm) or an increase of 40 bpm from baseline in the first 10 min of tilt, sustained for 2 min or more in the absence of hypotension (i.e., reduction in systolic blood pressure greater than 20 mmHg; Low et al. 2009). All participants included in this study met HUT criteria for POTS.

### Assessment of autonomic cardiovascular regulation

After meeting the eligibility criteria discussed above, each participant completed a pre-HIRREM visit for enrollment and data collection. Continuous BP and HR were acquired from noninvasive finger arterial pressure measurements and electrocardiogram for a minimum of 10 min with subjects lying down quietly, supine. Recordings were obtained at the baseline HIRREM enrollment visit, and a median of 8 days (range 0–32) after completion of the last HIRREM session. Systolic BP and beat-to-beat, RR intervals (RRI) files generated via the data acquisition system (BIOPAC acquisition system and Acknowledge 4.2 software, Santa Barbara, CA) at 1000 Hz were analyzed using Nevrokard BRS software (Nevrokard BRS, Medistar, Ljubljana, Slovenia), and analysis was conducted on the first complete 5-min epoch that was considered to be acceptable for analysis. For calculation of SDNN, the R–R intervals were visually inspected, and the data considered as artifact were manually removed. Spectral power was calculated with fast Fourier transform (FFT) with high-frequency (HF) power band set as (0.15–0.40 Hz) and low-frequency (LF) power band (0.04–0.15 Hz) on segment length 128 points, 50 % overlap. Recording periods of 256 s were analyzed, each yielding 512 data points after re-sampling at the 2-Hz frequency with the application of Hanning window. Spectral measures of BRS including low-frequency (LF) and high-frequency (HF) alpha indices were also calculated (Parati et al. 1995; Maestri et al. 2005).

### Self-reported nausea, dizziness, and autonomic symptoms

Subjects completed a Nausea/Dizziness Questionnaire which included 14 items related to nausea, dizziness, and autonomic regulation, rated on a Likert scale from 0 (none) to 4 (severe) for each item. Questionnaires were completed 1–2 weeks before HUT test and at the same visit as the post-HIRREM blood pressure recording.

### HIRREM process

Procedures for the provision of HIRREM have been discussed in detail previously (Gerdes et al. 2013). Prior to starting the HIRREM sessions, each participant had a baseline assessment to evaluate brain electrical frequencies and amplitudes. The HIRREM assessment (approximately 45 min) consisted of two-channel recordings of brain electrical activity from at least six paired locations on the scalp (F3/F4, C3/C4, T3/T4, P3/P4, FZ/OZ, and O1/O2) with HIRREM equipment (Brain State Technologies, Scottsdale, Arizona) that samples at 256 Hz, using a 16-bit A/D convertor with notch filter for rejection of signal >50 dB at 50 or 60 Hz, with signal processing performed on a 64-bit computer processor. Recordings were conducted at each location with eyes closed (1 min), eyes partially open to permit transition in arousal state (1 min), and eyes open (1 min while performing a mental task, e.g., recalling numbers and reading a passage). Data collected were subject to analysis in HIRREM software to permit visual and quantitative assessment of the relative symmetry between homologous brain regions, as well as the proportionation or distribution of amplitudes across different frequency bands at each location. Assessment data were then used to choose protocols for the initial HIRREM session. A typical HIRREM session (approximately 90 min) consisted of four to ten HIRREM protocols (6–30 min each), some done with eyes closed and some with eyes open, with the participant being asked to relax while sitting or reclining comfortably in a chair. Most protocols consisted of recording brain electrical activity through two channels, using sensors placed at homologous regions of the hemispheres, and analyzing rhythms using software designs that include proprietary mathematical algorithms, with occasional protocols entailing recording at a single cortical region with one channel (Gerdes et al. 2013). Software designs mirror cortical activity in specific frequency ranges, to support de-establishment of relatively invariant and potentially maladaptive activity patterns. Algorithms selected specific ranges of the brain electrical frequency spectrum, identified dominant frequencies in a middle range, and translated those frequencies to acoustic stimulation (auditory tones of variable pitch and timing) that was presented to participants through standard earphones (Creative EP-630 or Sony Stereo Headphones MDR-EX58V) with a delay of 8 ms at the lower limits. Volume (decibels) of acoustic stimulation was adjusted by each participant in accordance with their preference. Specific protocols for successive HIRREM sessions were chosen based on brain electrical data from the preceding session, which for purposes of technologist review was aggregated in broadband frequency bins (<1.0 Hz; 1.0–3.0 Hz; 3.0–5.5 Hz; 7.5–10.0 Hz; 10.0–12.0 Hz; 12.0–15.0 Hz; 15.0–23.0 Hz; 23.0–36.0 Hz; 36.0–48.0 Hz). Special attention was given to activity set points suggestive of dominant hemispheric asymmetries or proportionations of energy across the frequency spectrum considered to be potentially suboptimal (Gerdes et al. 2013). Participants generally completed two HIRREM sessions in a half day, separated by a 20- to 30-min break, and they received a median of 14 (10–16) sessions over 13 (8–17) days.

### Brain electrical activity changes

Temporal high-frequency (23–36 Hz) electrical asymmetry scores were calculated using 1-min epochs from the baseline assessment (T3/T4, eyes closed), and data from the penultimate minute of the penultimate HIRREM exercise at temporal locations (T3/T4, eyes closed), using the formula natural log (T4/T3) so that positive values indicate rightward dominance. The same data were also used to calculate values for the sum of temporal lobe high-frequency amplitudes (microvolts, µV) to explore for change in temporal lobe cortical arousal.

### Statistical analysis

Data are presented as mean percentage changes for autonomic cardiovascular regulation measures, mean values for symptom scores by individual item and the sum of all items, and median values for brain electrical activity. Changes in autonomic cardiovascular regulation measures, symptom scores, and sum of temporal lobe high-frequency amplitudes were evaluated using paired t tests. A correction strategy (e.g., Bonferroni) was not planned for the multiple comparisons entailed in analysis of the autonomic measures and symptom scores, because of likely correlation between the various measures and the exploratory nature of the study. Spearman’s correlation coefficient was used to estimate the correlation between baseline temporal lobe high-frequency electrical asymmetry and the change in asymmetry as measured at the final HIRREM session.

## Results

### Changes in autonomic cardiovascular regulation and self-reported symptoms

Table 1 shows changes in measures of autonomic cardiovascular regulation after subjects completed their HIRREM sessions. Data for SDNN, LF, and HF were not included for one subject who had frequent cardiac irregularities on the baseline assessment. Mean scores for each question of the Nausea/Dizziness Questionnaire, before and after HIRREM, are shown in Table 2. All subjects whose fludrocortisone had been discontinued were able to remain off it following HIRREM. No adverse events were reported, and there were no dropouts.

**Table 1:** Changes in measures of autonomic cardiovascular regulation, from baseline to after completion of HIRREM sessions

	Median value at baseline (range)	Median value after HIRREM (range)	Mean percentage change (range)	*p* value
LF alpha (ms/mmHg)	20 (12–43)	29.3 (13–34)	+28 (−27 to 127)	>0.2
HF alpha (ms/mmHg)	20.5 (12–53)	35.3 (17–77)	+65 (−6 to 180)	0.04
BRS sequence up (ms/mmHg)	16.6 (12–49)	25 (14–56)	+33 (−18 to 134)	0.18
BRS sequence down (ms/mmHg)	26.8 (12–40)	24.6 (12–63)	+54 (−63 to 260)	>0.2
BRS sequence all (ms/mmHg)	19 (12–47)	24.9 (15–61)	+37 (−40 to 177)	>0.2
SDNN (ms)	39 (35–92)	71 (44–120)	+51 (10 to 143)	0.03
LF (ms^2^)	854 (278–4552)	1167 (587–2326)	+43 (−73 to 167)	>0.2
HF (ms^2^)	917 (235–5204)	1605 (273–4769)	+107 (−32 to 372)	>0.2
MAP (mmHg)	79 (71–100)	79 (71–87)	−4 (−22 to 15)	>0.2
HR (beat/min)	65 (53–87)	73 (60–90)	+7 (−6 to 33)	>0.2

**Table 2:** Scores for self-reported intensity of nausea, dizziness, and general autonomic symptoms, on 0–4 Likert scale, before and after using HIRREM

	V1 mean (SD)	V2 mean (SD)	*p* value
Nausea	1.3 (0.8)	1.1 (0.9)	>0.2
Vomiting	0.4 (0.5)	0.0 (0)	0.08
Dizziness/lightheadedness	2.4 (1.0)	1.7 (0.8)	0.008
Passing out (syncope)	0.3 (0.8)	0.3 (0.8)	>0.2
Abdominal pain	1.6 (1.4)	1.4 (1.6)	>0.2
Constipation	0.7 (1.1)	0.4 (1.1)	0.17
Poor appetite	1.6 (1.4)	1.3 (1.3)	>0.2
Fatigue	2.6 (1.5)	1.7 (1.4)	0.08
Missing school	1.9 (1.3)	0.9 (1.2)	0.11
Feeling flushed, sweaty	1.4 (1.1)	0.7 (0.8)	0.14
Shortness of breath	1.3 (1.3)	1.0 (1.2)	>0.2
Chest pain	0.7 (1.3)	0.3 (0.5)	0.20
Heart racing	1.6 (1.5)	0.6 (1.1)	0.16
Headaches	2.3 (1.5)	1.7 (1.1)	>0.2

### Changes in temporal lobe high-frequency electrical activity

**Fig. 1:**
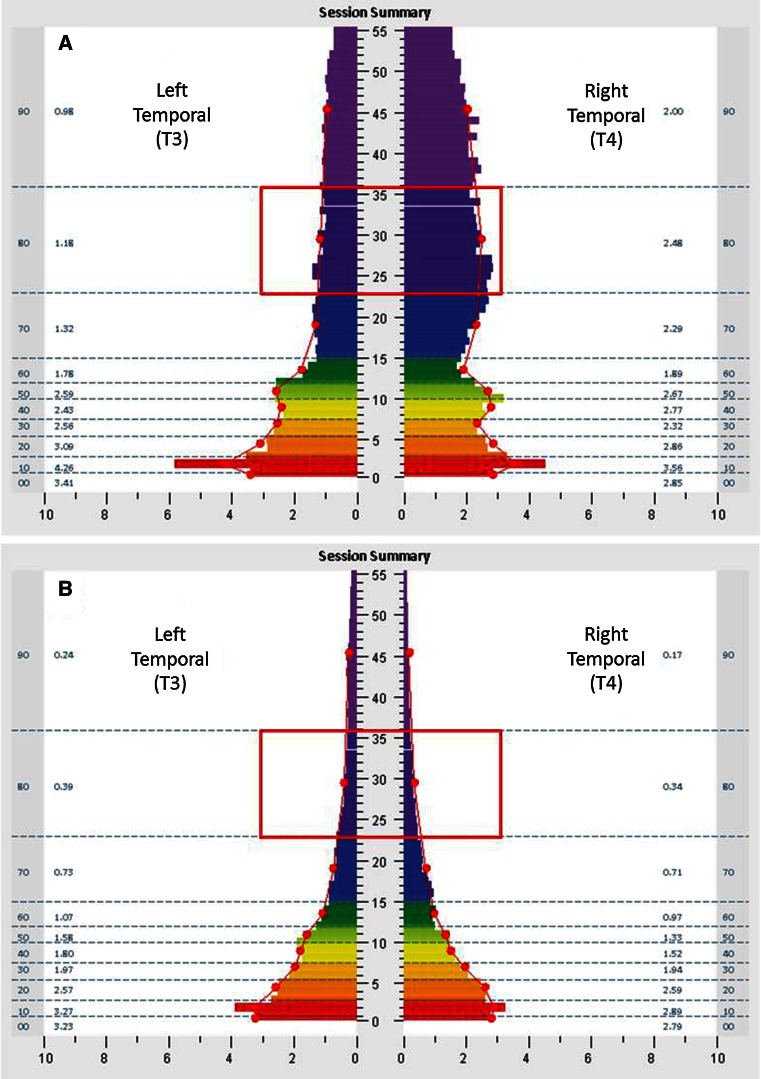
**a**, **b** FFT spectral display of electroencephalic data with frequency (Hertz, Hz, central *Y* axis) plotted against transformed amplitude (microvolts, µV, *X* axis). These graphs from a study subject represent 1 min of data from the T3/T4 montage with eyes closed (EC) at the baseline assessment (**a**) and at the penultimate minute of the penultimate session (**b**). *Columns* to the *left* and *right* denote ten frequency bands of aggregated data (00: <1.0 Hz; 10: 1.0–3.0 Hz; 20: 3.0–5.5 Hz; 30: 5.5–7.5 Hz; 40: 7.5–10.0 Hz; 50: 10.0–12.0 Hz; 60: 12.0–15.0 Hz; 70: 15.0–23.0 Hz; 80: 23.0–36.0 Hz; and 90: 36.0–48.0 Hz) and numerical values for averages in those ranges. Amplitudes in the 23–36 Hz range (*dark purple color*) are outlined by *red boxes*

**Fig. 2:**
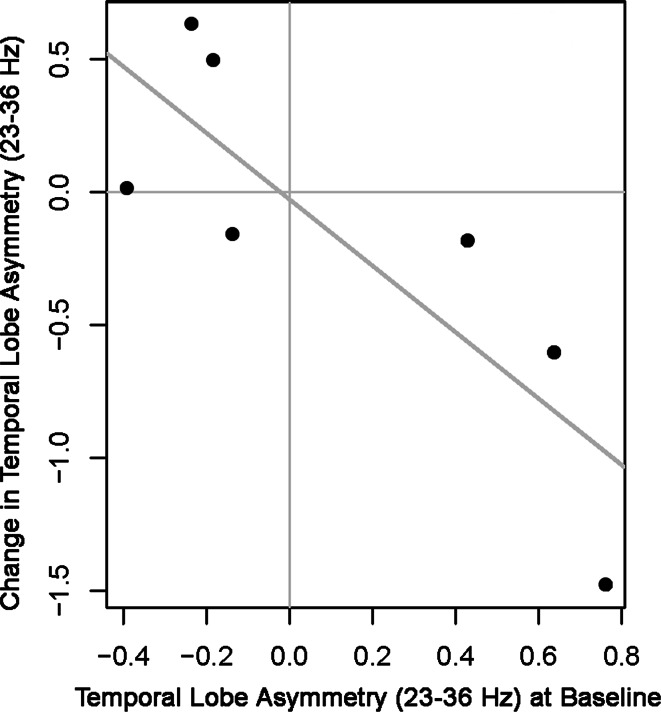
Change in asymmetry score for high frequency range (23–36 Hz) of brain electrical activity at bilateral temporal lobes (*vertical axis*), as a function of baseline asymmetry (*horizontal axis*)

Figure 1a, b shows spectrographs of brain electrical activity that typify shifts seen in temporal lobe brain electrical asymmetry in the high frequency range (and other ranges) over the course of HIRREM in one of the participants, a 15-year-old female. Relatively high amplitudes are seen especially in the high frequency ranges for both the left and right temporal lobes during the baseline assessment (a), and more so for the right. By the penultimate session (b), amplitudes are lower, and the two sides are relatively symmetrical. There was a negative correlation (Fig. 2) between baseline asymmetry score and the change in asymmetry score, calculated as final session asymmetry less baseline asymmetry (*ρ* = −0.89, *p* = 0.01). A statistically significant reduction was observed in the sum of the temporal high-frequency amplitudes (T3 plus T4) from baseline to the final session (median change = −3.8 µV, range = −17.8 to −0.7 µV, *p* = 0.02).

## Discussion

In this pilot study of adolescents with chronic nausea and POTS confirmed by abnormal HUT test, the use of HIRREM was associated with the increase in heart rate variability in the time domain (SDNN) and BRS in the frequency domain (HF alpha). Exploratory analyses of left and right temporal lobe electrical activity patterns showed reductions in both asymmetry and summed amplitudes in a high frequency (23–36 Hz) range. There were trends for reductions in self-reported nausea, dizziness, and autonomic symptoms, and those who were previously using fludrocortisone (four subjects) were able to discontinue that medication. The intervention was well tolerated, no adverse events occurred, and there were no dropouts.

Heart rate variability biofeedback may support symptom reduction and improved autonomic regulation in children with functional abdominal pain (Sowder et al. 2010), and therapeutic peripheral sensory stimulation (e.g., acupuncture) is reported to elicit reductions in abdominal symptoms, alterations in gastric slow waves, and increased heart rate variability (Liu et al. 2012; Witt et al. 2012; Zhang et al. 2015). The present report is consistent with these studies, and to our knowledge, it is the first to show that use of a nonpharmacological intervention is associated with increased heart rate variability and BRS, measures that are indicative of relative parasympathetic activation, in patients with POTS. Given that POTS is characterized by sympathetic bias (Shaltout et al. 2014) and that higher SDNN and HF alpha are indicative of relative parasympathetic activation, we infer that use of HIRREM in these participants facilitated more optimal autonomic functionality. Though all these approaches involve some degree of neocortical engagement, theoretically there may be advantages to interventions that can support the brain itself to optimize its activity, if it is possible to leverage its orchestrative role as the organ of central command, and in consideration of multiplier effects that can be realized through influence at higher levels of a system’s regulation.

The subjects’ changes in brain electrical activity patterns were consistent with previously reported observations of trends toward reduction in temporal high-frequency asymmetry and amplitudes among individuals enrolled in clinical trials of HIRREM for insomnia and menopausal hot flashes (Tegeler et al. 2012, 2015a; Gerdes et al. 2013). Increased high-frequency amplitudes have been implicated in hyperarousal associated with insomnia and menopausal hot flashes (Perlis et al. 2001; Campbell et al. 2011), and it would appear plausible that a decrease in temporal high-frequency amplitudes could also be consequential for some symptomatology associated with POTS. Preliminarily, we interpret the changes in temporal lobe high-frequency asymmetry and amplitudes to be consistent with a model of technology-assisted brain self-optimization, wherein the brain is facilitated through HIRREM to update itself with respect to its neural oscillatory patterns at key regions of the cortex. Iterated self-updating may support the brain to make self-adjustments to activity patterns including mechanisms for autonomic regulation.

The idea that “continual updating of knowledge [information] stores” (Sterling 2014) could be a basis for principled therapeutics is one of the tenets of the advanced model of physiological regulation known as *allostasis* (Sterling 2004, 2012), or “stability through change.” It has been proposed that allostasis has the potential to replace the conventional model of homeostasis (“stability through constancy”) through its definition of health as a state of *optimal predictive fluctuation* and its recognition, consistent with evolutionary theory, that fitness for life requires flexible adaptability of an organism’s operational set points across various system functionalities. Under allostasis, the brain is understood to serve as the master organ of central command, allocating resources so as to optimize responsivity to complex environmental contexts.

When the brain perceives the environment in a way that drives it to alter various system set points (e.g., increased sympathetic arousal related to chronic psychosocial stressors), neuroplasticity presents the potential for these set points to become dominant, with implications for functionality of any or all organ systems that are subject to neural regulation. An allostatic view of health implies that disease states can be expressed that are not the consequence of any intrinsic malfunctioning in biochemical or physiological mechanisms. Relatedly, in its recognition of the brain as the organ of central command, the allostasis concept may explain the comorbidity of psychological and somatic symptoms demonstrated in POTS and other autonomic disorders (Benarroch 2012; Tarbell et al. 2014). Furthermore, allostasis invokes the need for therapeutics that facilitate the brain’s natural learning capacities and dynamic responsivity to the full range of ambient signals, as opposed to the clamping of system set points toward standardized response levels (Sterling 2014).

The findings of the present study suggest that an allostatic neurotechnology—one that is brain-centric, change-oriented, and intended for purposes of self-optimization—merits further investigation as a means to support improved autonomic regulation through endogenous brain processes. Though physical mechanisms through which HIRREM may facilitate auto-calibration of neural oscillations are unknown, it is our conjecture that every individual has unique templates for his or her own patterns of brain activity as measured in the frequency domain—no less than they have unique fingerprints—and that any given individual oscillatory pattern is subject to disturbance for a myriad of reasons. On the basis of thermodynamic physical law whereby energy is neither created nor destroyed, it is plausible that presentation of serial acoustic tones that are resonant with dominant and changing brain frequencies may create opportunities for accretion or dissipation of neural energy in directions that are aligned with recovery of an individual’s own unique oscillatory templates.

This study was exploratory, and our inferences regarding the present findings are limited by the modest number of subjects. Because there was no active control group, we cannot rule out the possibility that some component of the results might be due to subjective expectation or “placebo responding.” We are not aware of data to suggest that subjective expectation alone could produce changes in both heart rate variability and temporal lobe electrical activity, of the magnitude demonstrated. Recently, it has been reported, for example, that patients with POTS who received an oral placebo demonstrated no change in heart rate beyond that attributable to background cardiovascular variability (Nwazue et al. 2014), though it is also important to consider that the HIRREM intervention entails greater subjective engagement than ingestion of a substance. Future studies may be designed to incorporate control interventions that entail alternative allostatic strategies (behavioral interventions) to evaluate the relative effect size associated with HIRREM, and studies with larger sample sizes and longer follow-up may help ascertain whether baseline asymmetry contributes to clinical improvement, as well as the duration of measured benefits. Furthermore, the use of more detailed and validated symptom questionnaires to assess symptoms at baseline as well as longitudinally will be essential to more objectively define the impact of treatment strategies such as HIRREM.

In conclusion, the present study showed that in a case series of adolescents with POTS, use of a closed-loop acoustic stimulation intervention, HIRREM, was associated with improved autonomic regulation, reduction in temporal high-frequency electrical asymmetry and amplitudes, and a trend toward reduction in autonomic symptomatology. The findings suggest it may be possible to impact peripheral autonomic physiology through noninvasive interaction with lateralized activity patterns at the level of the cerebral cortex. Given the absence of evidence-based therapies for orthostatic intolerance associated with nausea, and side effects typically associated with pharmacological strategies directed at the autonomic nervous system, further study of allostatic neurotechnology such as HIRREM appears warranted.

## Abbreviations

ANS: Autonomic nervous system
BRS: Baroreflex sensitivity
HF: High frequency
HR: Heart rate
HRV: Heart rate variability
Hz: Hertz
HIRREM: High-resolution, relational, resonance-based, electroencephalic mirroring
LF: Low frequency
µV: Microvolts
HUT: Head upright tilt test
POTS: Postural orthostatic tachycardia syndrome
SD: Standard deviation
SDNN: Standard deviation of the normal-to-normal interval
